# Cultural adaptation, translation and validation of the Spanish version Debriefing Experience Scale

**DOI:** 10.1371/journal.pone.0267956

**Published:** 2022-05-05

**Authors:** Mariona Farrés-Tarafa, David Bande Julian, Urbano Lorenzo-Seva, Barbara Hurtado-Pardos, Marta Raurell-Torredà, Irma Casas, Jaime Carballedo-Pulido, Juan Roldán-Merino

**Affiliations:** 1 Campus Docent, Sant Joan de Déu—Fundació Privada, School of Nursing, University of Barcelona, Barcelona, Spain; 2 Research Group GIES (Grupo de Investigación en Enfermería, Educación y Sociedad), Barcelona, Spain; 3 Member Research Group GRISIMula (Grupo Emergente 2017 SGR 531, Grupo en Recerca Enfermera en Simulación), Barcelona, Spain; 4 Anesthesiologist, Servicio Anestesiología, Reanimación y Tratamiento del dolor, Parc de Salut Mar, Barcelona, Spain; 5 Universitat Rovira I Virgili, Tarragona, Spain; 6 Member Research Group GRIN (Grupo Consolidado de Recerca Infermeria, SRG:664), Barcelona, Spain; 7 Universidad de Barcelona, Barcelona, Spain; 8 President Research Group GRISIMula (Grupo Emergente 2017 SGR 531, Grupo en Recerca Enfermera en Simulación), Barcelona, Spain; 9 Universitat Autònoma de Barcelona, Barcelona, Spain; 10 Preventive Medicine Service, Hospital Germans Trias i Pujol, Barcelona, Spain; 11 Research Group Innovation in Respiratory Infections and Tuberculosis Diagnosis (Group Consolidat 2017 SGR 494), Barcelona, Spain; 12 Research Group GEIMAC (Consolidated Group 2017–1681: Group of Studies of Invarianza of the Instruments of Measurement and Analysis of Change in the Social and Health Areas), Barcelona, Spain; Murcia University, Spain, SPAIN

## Abstract

Clinical simulation as a teaching methodology allows the student to train and learn technical abilities and/or non-technical abilities. One of the key elements of this teaching methodology is the debriefing, which consists of a conversation between several people, in which the participants go over a real or simulated event in order to analyze their actions and reflect on the role that thought processes, psychomotor skills and emotional states can play in maintaining, or improving their performance in the future. The Debriefing Experience Scale allows the experience of students in debriefing to be measured. The objective of this study is to translate the Debriefing Experience Scale (DES) into Spanish and analyze its reliability and validity to measure the experience of nursing students during the debriefing. The study was developed in two phases: One: the adaption of the instrument to Spanish, two: a transversal study carried out in a sample of 290 nursing students. The psychometric properties were analyzed in terms of reliability and construct validity using confirmatory factorial analysis (CFA). Cronbach’s alpha was adequate for all the scales and for each one of the dimensions. The confirmatory factorial analysis showed that the 4-dimensional model is acceptable for both scales (experience and opinion). The Spanish version Debriefing Experience Scale questionnaire is useful, valid and reliable for use to measure the debriefing experience of university students in a simulation activity.

## Introduction

In health science studies in order to complete the proposals generated by the Bologna process new methodologies have been developed based on active participation by the student, amongst them clinical simulation.

Clinical simulation as a teaching methodology allows the student to train and learn technical abilities and/or non-technical abilities such as: communication; leadership; teamwork; situational awareness; decision-making; resource management; safe practice; adverse event minimization and mitigation; and professionalism [1], through reflective learning, which facilitates critical thinking in a safe environment, without risk to patients or the participants.

One of the key elements of this teaching method is the debriefing. Existing research [2, 3] provides evidence that the debriefing is the most important component of the learning process of any experience based on simulation [4]. It has to be planned and directed by a facilitator (debriefer) who orients the discussion from reflection, focusing on the learning objectives and on the application of knowledge. Maestre and Rudolph (2015) define it as a conversation between various people, who go over a real or simulated event, in which the participants analyze their actions and reflect on the role that thought processes, psychomotor skills and emotional states can play in maintaining or improving their performance in the future [5].

There are many definitions of debriefing, they all agree that it is the sum of feedback plus reflection on an experience [6], carried out through means of analysis of the thought process, guided by action and decision making during the simulation (what was done, why it was done, what could have been done differently) in order to apply the results obtained to future situations [3, 5, 7–10]. When the instructions encourage a high level of commitment from the participants, they have better retention and undergo deeper learning, raising the probabilities that new or reinforced knowledge, abilities and attitudes will be transferred to clinical practice, or better health performance in general [11].

Although there are different styles of debriefing, they all share characteristic defining elements and a structure, which is generally divided into three phases: 1)1a. Emotions and reactions, 1b description and summary, 2) analysis and 3) closure and conclusions [12]. The first phase of reactions takes place immediately after finishing the simulation experience, when the participants meet with the facilitator. It is a phase of emotional discharge which allows the transition which makes the reflexive part possible. Phase 1b. consists of constructing the reality lived and shared together with each of the participants, according to the perceptions of each individual. The second phase is an analysis phase, when the cognitive and learning processes take place for each participant. The goal is to discuss the pre-established and emerging learning objectives, to examine the thought processes of the participants more deeply, diagnose the participants’ position regarding the objectives they are trying to reach and reflect on how to improve when putting them into practice in the future and in different contexts. Lastly, before finishing the debriefing, there is a third phase of closure and conclusions, which has the objective of presenting the results of the learning through a process of refection and verbalization of what was learned, which makes better integration possible.

In the literature there are numerous studies that emphasize the role of the debriefer in the debriefing, however, there is still little known about how the participants experience a debriefing session in order to provide an understanding of the expected learning process occurring while it takes place [13, 14]. Because of this in 2012 Shelly J. Reed developed a questionnaire to find out how students experience debriefing: the Debriefing Experience Scale (DES). This 20 item questionnaire has been translated into Norwegian and Portuguese and validated [15, 16]. Both studies concluded that it is a useful, valid and reliable questionnaire, used so participants can evaluate the simulation experience. They suggest that it should be validated in more nursing programs in different cultural contexts.

The objective of this study was to translate the Debriefing Experience Scale (DES) to Spanish, and analyse its reliability and validity for finding out about nursing students’ experiences during debriefing.

## Methods

### Design

The study was designed in two phases: the first implies the translation and adaptation of the questionnaire and the second consists of validation using a transversal design.

### Debriefing Experience Scale

The Debriefing Experience Scale was developed by Reed in the United States with the aim of measuring the experience of students during debriefing [14]. The questionnaire is configured with 20 items, grouped in four dimensions. It can be completed in approximately 12 minutes. The dimensions correspond to: D1 learning and making connections; D2. analysing ideas and feelings; D3. the ability of the facilitator in directing the debriefing; and D4. appropriate guidance from the facilitator.

The same scale allows each item to evaluate the opinion of the students of their experience in the debriefing, and on the other hand the experience based on the importance it has for each student.

Each item is evaluated using a Likert scale with 5 possible replies, which are:

1) strongly disagree, 2) disagree, 3) undecided, 4) agree, and 5) strongly agree.

The sum of the scores for all the items for each dimension gives us an estimation of the experience of the students in the debriefing and the importance the experience has for them.

The DES questionnaire has demonstrated good and moderate validity in the nursing student population in the United States of America and a reliability of .93 (experience) and .91 (importance).

### Cultural and linguistic adaptation of the Debriefing Experience Scale

The cultural adaption and translation of the questionnaire was carried out in several stages and in agreement with standardized criteria [17]. In the first stage, two translators translate the English to Spanish (t1 and t2). In the second stage both versions, t2 and t2 are merged. In this stage a research team made up of two clinical simulation teachers, accredited by the *Boston Children’s Hospital*, *Simulator Program*, Boston; two teachers expert in psychomotor skills and three nurses with experience of advanced clinical practice. The research team resolved any discrepancies, thereby obtaining the version t3. In the third stage, the t3 version was translated from Spanish to English by two native English translators (t4 and t5). The research team checked both translations and compared them with the original. All the researchers agreed that the items in the Spanish version coincided with the original English version. However, to obtain the best degree of semantic equivalence, the committee of experts decided to modify “Aprender y hacer conexiones” to “Aprender y relacionar conceptos” in dimension 1, so they also modified item 1 “Aprender y hacer conexiones” to “Aprender y relacionar conceptos”. In item 8 they modified “hacer conexiones de la teoría con situaciones de la vida real” to “me ayudó a relacionar la teoría con situaciones de la vida real”. In item 10 they modified “equipo sanitario” to “equipo” and in item 11 “psíquicamente cómodo” to “psicológicamente seguro”.

Afterwards, in the fourth stage, version t6 was tested on a small sample of students (n = 30), who concluded that it was easy to understand and needed little time to complete; approximately 12 minutes. Table 1 shows the semantic equivalents of the original and the version adapted to Spanish.

**Table 1:** Shows the semantic equivalence of items from English to Spanish that were psychometrically validated.

	English	Spanish
D1.	**Learning and making connections**	**Aprender y relacionar conceptos**
**Item 1**	Debriefing helped me to make connections in my learning	El debriefing me ayudó a relacionar conceptos en mi aprendizaje
**Item 2**	Debriefing was helpful in processing the simulation experience	El debriefing fue útil para procesar la experiencia de simulación
**Item 3**	Debriefing provided me with a learning opportunity.	El debriefing me proporcionó una oportunidad de aprendizaje
**Item 4**	Debriefing helped me to find meaning in the simulation.	El debriefing me ayudó a encontrarle sentido a la simulación
**Item 5**	My questions from the simulation were answered by debriefing.	Mis preguntas generadas en la simulación se resolvieron en el debriefing
**Item 6**	I became more aware of myself during the debriefing session	Tomé más consciencia de mí mismo durante la sesión del debriefing
**Item 7**	Debriefing helped me to clarify problems.	El debriefing me ayudó a clarificar dudas
**Item 8**	Debriefing helped me to make connections between theory and real-life situations.	El debriefing me ayudó a relacionar la teoría con situaciones de la vida real
**D2.**	**Analyzing thoughts and feelings**	**Analizar ideas y sentimientos**
**Item 9**	Debriefing helped me to analyze my thoughts.	El debriefing me ayudó a analizar mis ideas
**Item 10**	The facilitator reinforced aspects of the health care team’s behavior.	El facilitador reforzó aspectos del comportamiento del equipo
**Item 11**	The debriefing environment was physically comfortable.	El ambiente del debriefing era psicológicamente seguro
**Item 12**	Unsettled feelings from the simulation were resolved by debriefing.	Los sentimientos inquietantes de la simulación se resolvieron durante el debriefing
**D3.**	**Facilitator skill in conducting the debriefing**	**La habilidad del facilitador dirigiendo el debriefing**
**Item 13**	The facilitator allowed me enough time to verbalize my feelings before commenting.	El facilitador me dejó suficiente tiempo para verbalizar mis sentimientos antes de hacer los comentarios
**Item 14**	The debriefing session facilitator talked the right amount during debriefing	El facilitador de la sesión de debriefing habló la cantidad adecuada durante el debriefing
**Item 15**	Debriefing provided a means for me to reflect on my actions during the simulation.	El debriefing me proporcionó un medio para reflexionar sobre mis acciones durante la simulación
**Item 16**	I had enough time to debrief thoroughly.	Tuve suficiente tiempo para realizar el debriefing concienzudamente
**Item 17**	The debriefing session facilitator was an expert in the content area	El facilitador de la sesión de debriefing era experto en el área de contenido
**D4.**	**Appropriate facilitator guidance**	**Guía Apropiada del facilitador**
**Item 18**	The facilitator taught the right amount during the debriefing session.	El facilitador enseñó la cantidad correcta durante la sesión de debriefing
Item 19	The facilitator provided constructive evaluation of the simulation during debriefing.	El facilitador proporcionó una evaluación constructiva de la simulación durante el debriefing
Item 20	The facilitator provided adequate guidance during the debriefing.	El facilitador guio adecuadamente el debriefing

Finally the questionnaire was administered to the undergraduate nursing students included in the sample to analyze the psychometric properties of the Spanish version of the Debriefing Experience Scale questionnaire.

### Participants and setting

The study sample consisted of 290 undergraduate nursing students registered on the 2019–2020 academic course. A convenience sample was carried out. All students who gave their consent to participate in the study, and had also taken part in a clinical simulation during the course, were included. Only those students who were not present at the time of administering the questionnaire were excluded. The sample size was calculated based on internal consistency and construct validity. The recommendations of Streiner, Norman & Cairney, were followed to estimate the internal consistency; they consider that between 5–20 individuals should be included for each item making up the questionnaire [18]. In this study, it was agreed to include a minimum of 15 individuals per item. Additionally, for the construct validity, Kline (2015) established that the minimum number of participants necessary to realize a confirmatory factorial analysis (CFA) should be 250 students [19].

### Variables and source of information

All items related with the Debriefing Experience Scale questionnaire were collected as variables. Other sociodemographic variables, such as: age, sex, academic year, average grade on their transcript for the previous course, teaching shift, whether they were currently working, if they worked in healthcare, type of contract and working shift were also collected.

### Statistical analysis

The reliability of the questionnaire was measured using the Cronbach’s alpha coefficient and values considered acceptable were those over.70 [20, 21].

To analyze the construct validity a confirmatory factorial analysis (CFA) was carried out using maximum likelihood estimation. The goodness of fit of the model was evaluated from various indices: the normalized chi-square, defined as the ratio of the chi-square value to the number of degrees of freedom (χ2/df); the adjusted Goodness of Fit Index (AGFI); Goodness of Fit Index (GFI); Comparative Fit Index (CFI); Bentler Bonnet Non-Normed Fit Index (BBNNFI); Bentler Bonnet Normed Fit Index (BNNFI); Root Mean Standard Error of Approximation (RMSEA); Root Mean Square Residual (RMR) and the Standardized Root Mean Squared Residue (SRMR). To consider a good global fit the criteria adopted was the obtention of the folloiwng adjustment value: X^2^/df values between 2 and 6 [22]; AGFI, GFI, CFI, BBNNFI y BBNFI values greater than or equal to .90.

For the RMSEA, RMR and SRMR indices, values equal to or less than .05 were considered excellent, while those over .05 and equal to or lower than .08 were acceptable [23–25].

CFA models were estimated using structural equation modeling (EQS 6.4 for Windows, Multivariate Software, Inc., Encino, CA, USA).

A descriptive analysis was carried out using frequencies and percentages, measures of central tendency and dispersion. Data analyses were performed using SPSS for Windows 27 (SPSS Institute, Chicago, IL, USA).

### Ethical considerations

The study was approved by the Clinical Investigation Ethics Committee of the San Joan de Déu Foundation, with the assigned code CEIC PIC-42-19. All the participants gave their written consent to voluntarily participate in the study, having been informed of the aim of the research. The permission of the author was also obtained for the translation and adaption of the instrument to Spanish.

## Results

### Demographic characteristics

A total of 290 nursing students took part, with an average age of 22.9 (SD 5.4), and 85.5% were women. The average grade on their transcript for the previous academic course was 8.0 (SD 0.5). 56.2% of the students were registered in the group doing classes in the mornings. Half of the students declared they were working at the time (50.3%); and of these 41.8% had a permanent contract and 37.6% of the students working, were doing so in the healthcare sector (Table 2).

**Table 2:** Sociodemographic characteristics of the study population (n = 290).

	n	%
Age (SD)	22.9 (SD 5.8)	
Average grade previous course (SD) (n = 250)	8.0 (SD 0.5)	
Sex		
Women	248	85.5
Men	42	14.5
Study schedule.		
Morning	163	56.2
Afternoon	127	43.8
Academic course		
Second	143	49.3
Third	76	26.2
Fourth	71	24.5
Currently employed		
Yes	146	50.3
No	144	49.7
Type of contract		
Permanent employment	61	21.0
Temporary employment	85	29.3
Working in healthcare sector		
Yes	108	37.2
No	38	13.1

### Reliability

The Cronbach’s alpha or coefficient of internal consistency for the total of the Debriefing Experience Scale for experience and for importance of simulation in the questionnaire was .926 and .933 respectively (Table 3).

**Table 3:** Internal consistency coefficient Cronbach’s alpha for Debriefing Experience Scale.

Item contents summarized	Cronbach’s alpha	
	Experience	Importance in simulation
**D1.** Learning and making connections	.890	.864
**D2.** Analyzing thoughts and feelings	.725	.788
**D3.** Facilitator skill in conducting the debriefing	.792	.800
**D4.** Appropriate facilitator guidance	.750	.765
Total scale	.926	.933

### Construct validity

#### Confirmatory factorial analysis (CFA)

The parameters were estimated using the maximum likelihood estimation method. A 4-dimensional model was proposed, identical to the structure of the original version of the questionnaire, with the aim of checking if the model was adequate.

The chi-squared test was statistically significant, although the adjustment ratio was 2.1 (experience) and 2.8 (importance), however, between 2 and 6 is considered reasonably good. The rest of the indices analyzed showed the same tendency, so we can conclude that the model fits correctly (Table 4).

**Fig 1:**
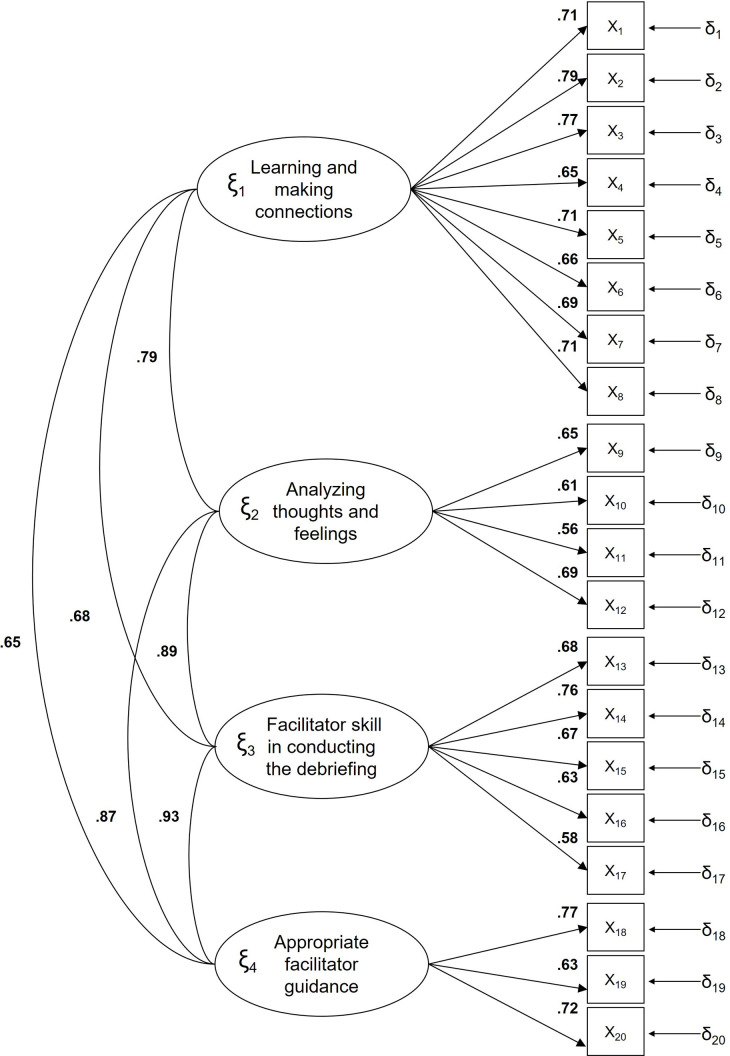
Standardized model parameters for the Debriefing Experience Scale for experience of simulation.

**Fig 2:**
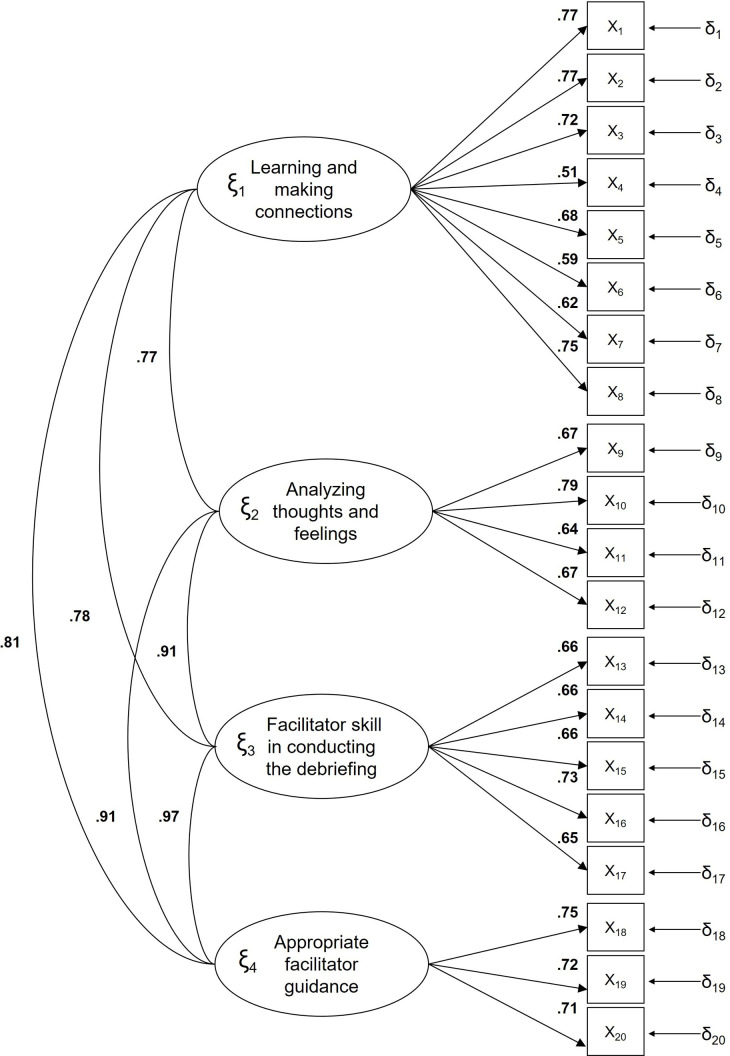
Standardized model parameters for the Debriefing Experience Scale for importance of simulation.

All saturations were over 0.50. The correlations between the factors of experience and importance of the debriefing were high (Figs 1 and 2 respectively).

**Table 4:** Indices of goodness of fit of the confirmatory model the Spanish version Debriefing Experience Scale.

	Evaluate the debriefing experience for the student	
	Student experience	Importance in Debriefing
**INDEX**	**VALUE**	**VALUE**
BBNFI	.876	.848
BBNNFI	.919	.877
CFI	.930	.894
GFI	.894	.860
AGFI	.864	.821
RMR	.018	.016
RMSR	.048	.050
RMSEA	.062 (90% CI:.053 -.071)	.080 (90% CI: .072 - .089)
α Cronbach	.926	.933
Goodness of fit test	χ^2^ = 348.523; gl = 164; *P* < .0001	χ^2^ = 470.658; gl = 164; *P* < .0001
Reason for fit	χ^2^ / gl = 2.1 between 2–6	χ^2^ / gl = 2.8 between 2–6

BBNFI: Bentler Bonnet Normed Fit Index. BBNNFI: Bentler Bonnet Non-Normed Fit Index. CFI: Comparative Fit Index. GFI: Goodness of Fit Index. AGFI: Adjusted Goodness of Fit Index. RMR: Root Mean Square Residual. RMSR: Root Mean Standard Error Standardized. RMSEA: Root Mean Standard Error of Approximation

## Discussion

The aim of the study was to adapt the Debriefing Experience Scale (DES) to Spanish and evaluate the psychometric properties in nursing students in Spain. The instrument was developed to evaluate the experience and the importance of debriefing in simulation. The results obtained in this study show that the Spanish version of the Debriefing Experience Scale have adequate psychometric properties in terms of internal consistency and construct validity.

It consists of 20 items grouped in four dimensions, which aim to evaluate the experience and importance of the simulation debriefing for the nursing students.

The reliability of the questionnaire was adequate, it obtained a Cronbach’s alfa over .70 for the whole questionnaire and for each of the dimensions of both subscales (experience and importance). The highest value was obtained for dimension D1 Learning and making connections. For the rest of the dimensions (D2. Analyzing thoughts and feelings, D3. Facilitator skill in conducting the debriefing and D4. Appropriate facilitator guidance) the alpha varied between .725 and .800. This instrument has been translated into different languages in different countries (Norwegian and Portuguese) and these studies reported similar values to those found in our study [15, 16].

The original scale, created by Reed was validated in a sample of 130 nursing students in the USA and obtained a Cronbach’s alpha for the total questionnaire, experience and importance of the debriefing of .930 and .910 respectively [Reed 2012]. The alpha values for each of the dimensions varied between .650 and .890, the highest being for dimension D1 Learning and making connections, as found in our study. The instrument translated into Portuguese [16] was validated in a sample of 103 nursing professionals and obtained similar results to our study and the original validation of the study (.940 for experience and .96 for importance of debriefing in simulation). However, the instrument translated to Norwegian [15], with a sample of 146 nursing students, obtained values of the total scale of .860 and .640 for experience and importance respectively.

The CFA of this study showed an adequate fit for the structure of 4 factors, consistent with the original version [14].

In our study a CFA was carried out using the maximum likelihood estimation method to determine whether the scores reproduced the structure of 4 dimensions, on which the original version is based. The results obtained for the indices used to carry out the factorial validity and the goodness of fit were acceptable assuming an adequate model fit.

The authors of the Norwegian translation and validation [15], decided to withdraw item 12 “Unsettled feelings from the simulation were resolved by debriefing” and item 13 “The facilitator allowed me enough time to verbalize my feelings before commenting”, obtaining a scale of 18 items instead of 20 and a Cronbach’s alpha for the total questionnaire of .91. Furthermore, using the exploratory factorial analysis (Keiser), pattern matrix and structure matrix, they proposed reducing the dimensions of the instrument to two dimensions “the experience of learning in debriefing” and “the facilitator’s ability in conducting debriefing”, however, as the first dimension explains the main part of the total variance, it could justify a scale of just one dimension. The Portuguese version of the questionnaire by Almeida et al. using exploratory factorial analysis (octagonal rotation) obtained unexpected results, with a model with three dimensions, despite this they decided to maintain the four dimensions of the original instrument and justify the unexpected fit with the sample taken, as they were not student nurses, but professionals [16].

### Limitations

Our study has certain limitations. In the first place, we selected a convenience sample from only one university in Barcelona, so it is possible that our results cannot be generalized to all the nursing students. However, the sociodemographic and labor characteristics of the students in this study are similar to those in other universities in Spain.

A limitation that should be considered is that how the simulation is implemented could differ between institutions and countries, which could cause differences in the way the concepts used in the questionnaire are interpreted. However, to implement the simulation for this study the Standard VI: the Debriefing Process of the International Nursing Association for Clinical Simulation was used [26].

## Conclusions

The Spanish version of the Debriefing Experience Scale (DES-sp) questionnaire is a valid and applicable instrument to find out about the debriefing experience of university students. The scale evaluated two aspects: experience of the debriefing and opinion of the debriefing in the simulation. It is a scale that requires very little time to be self-completed.

## Supporting information

S1 Data (XLSX)
